# Factors Related to the Rise of Congenital Syphilis From the Perspectives of Prenatal Providers and Birthing Parents in Chicago, IL, USA

**DOI:** 10.1093/ofid/ofae595

**Published:** 2024-10-08

**Authors:** John M Flores, Nikki Kasal, Caroline Montag, Alicia Dawdani, Ellen Almirol, Jackson M C Montgomery, Daniela Zimmer, Jessica Ridgway, John A Schneider

**Affiliations:** Department of Medicine, University of Chicago, Chicago, Illinois, USA; Department of Pediatrics, University of Chicago, Chicago, Illinois, USA; Pritzker School of Medicine, University of Chicago, Chicago, Illinois, USA; Pritzker School of Medicine, University of Chicago, Chicago, Illinois, USA; Chicago Center for HIV Elimination, University of Chicago, Chicago, Illinois, USA; Chicago Center for HIV Elimination, University of Chicago, Chicago, Illinois, USA; Chicago Center for HIV Elimination, University of Chicago, Chicago, Illinois, USA; Department of Medicine, University of Chicago, Chicago, Illinois, USA; Department of Medicine, University of Chicago, Chicago, Illinois, USA; Chicago Center for HIV Elimination, University of Chicago, Chicago, Illinois, USA; Department of Medicine, University of Chicago, Chicago, Illinois, USA; Chicago Center for HIV Elimination, University of Chicago, Chicago, Illinois, USA; Department of Public Health Sciences, University of Chicago, Chicago, Illinois, USA

**Keywords:** congenital syphilis, maternal–fetal medicine, qualitative study, sexually transmitted infections, syphilis

## Abstract

**Background:**

Rates of congenital syphilis (CS) in the United States have risen sharply in recent years. There has been sparse research centering the voices and experiences of birthing parents who have delivered infants with CS and prenatal providers in Chicago or the surrounding Midwestern United States to date. Additionally, there has been little research on the role of extrinsic factors, such as stigma and attitudes surrounding CS in an individual's community, in their entry into and retention in the CS prevention cascade.

**Methods:**

Semistructured interviews seeking to gather perspectives and factors related to the rise of CS were conducted with birthing parents who delivered infants with CS at a major academic medical institution (AMI) and the prenatal providers who served them. This was supplemented by retrospective data of birthing parent outcomes.

**Results:**

Barriers elicited during the interviews included insufficient penicillin uptake, limited transportation, poor communication between providers and patients, gaps in patient understanding or knowledge around CS contraction and treatment, missed appointments, appointment burden for patients, life stressors of patients, housing instability, childcare difficulties, and stigma related to the CS diagnosis. The quantitative data revealed differing proportions of CS outcomes and care between those with care within the AMI, those with care outside the AMI, and those who had no prenatal care.

**Conclusions:**

This study found numerous perspectives and factors that may explain the rise of CS along various stages of the syphilis care continuum through in-depth interviews of prenatal providers and birthing parents.

## Teaser

This study found numerous perspectives and factors that may explain the rise of congenital syphilis (CS) along various stages of the syphilis care continuum through in depth interviews of prenatal providers and birthing parents of infants with CS.

Rates of congenital syphilis (CS) in the United States have risen sharply in recent years. In 2022, the Centers for Disease Control and Prevention (CDC) reported 3755 cases of CS nationwide, representing a 183% increase since 2018 [1]. In the city of Chicago, the total number of reported CS cases increased by >400% between 2019 and 2022 alone [2]. This rise has largely coincided with the nationwide resurgence of syphilis among reproductive-age cisgender women, for whom rates of primary and secondary syphilis increased by 676% from 2012 to 2021 [3]. Additional racial and ethnic disparities in the incidence of CS as well as primary and secondary syphilis further worsen the structural inequality amidst this growing epidemic [4, 5]. According to the 2019 Global Burden of Disease Study, the global number of incident syphilis cases increased from 8.8 million in 1990 to 14.1 million in 2019. The age-standardized incidence rate (ASIR) also rose from 160.03 per 100 000 persons to 178.48 per 100 000 persons during the same period. This increase was particularly notable in regions with high and high-middle sociodemographic indices and among males, while the incidence among females decreased. Further data from the Global Burden of Disease Study 2021 show that in 2021 the global prevalence of syphilis was 70.5 million cases [6, 7]. According to a 2019 study, the estimated global rate of congenital syphilis in 2016 was 473 cases per 100 000 live births, resulting in ∼661 000 total cases of congenital syphilis. This includes 355 000 adverse birth outcomes such as early fetal deaths, stillbirths, neonatal deaths, preterm or low-birthweight births, and infants with clinical congenital syphilis [8].

CS can cause significant infant morbidity and mortality if untreated, including but not limited to deafness, neurological impairment, bone deformities, stillbirth, and neonatal death [9]. Fortunately, it is highly preventable through early detection and treatment. The American College of Obstetrics and Gynecology (ACOG) has recently issued updated guidelines for syphilis screening for pregnant patients, with recommended universal syphilis screening at the first prenatal visit in the first trimester, followed by universal rescreening in the third trimester and at delivery [10]. In patients who test positive for syphilis during pregnancy, timely treatment with benzathine penicillin G reduces the risk of CS by 97% and stillbirth by 82% [11].

Despite the efficacy of penicillin in preventing CS, there are several distinct linkage gaps in the CS prevention cascade that can preclude pregnant patients from receiving timely syphilis testing and treatment. According to the CDC, 88% of birthing parents of infants with reported CS in 2022 received either no or nontimely syphilis testing or no or inadequate treatment during pregnancy, the former of which was also highly associated with no prenatal care [3]. Timely testing did not necessarily equate to adequate treatment: in fact, only 7% of birthing parents for whom timely testing and diagnosis occurred received adequate treatment before delivery [3].

Previous qualitative research featuring the perspectives of pregnant or postpartum birthing parents and prenatal providers has identified several factors underlying these missed prevention opportunities, including health insurance limitations leading to no or delayed prenatal care, transportation, and other social factors causing loss of patients to follow-up, economic instability/cost of treatment, and lack of patient knowledge and understanding about CS [12–17]. Studies from the Western and Southern United States also acknowledged the impact of context-dependent factors on gaps in CS prevention, suggesting possible geographic variation in findings [12, 13, 15].

Previous research, through a retrospective chart abstraction of electronic health records (EHRs), has described factors associated with maternal and fetal syphilis diagnoses at a Chicago-area hospital, including a history of maternal psychiatric illness [5]. There has been sparse research centering the voices and experiences of birthing parents who have delivered infants with proven or possible CS in Chicago or the surrounding Midwestern United States to date. Additionally, there has been little research on the role of extrinsic factors, such as stigma or attitudes surrounding CS in an individual's community, in their entry into and retention in the CS prevention cascade. A previous study showed an association between HIV-related stigma and low adherence to antiretroviral medications and usage of health and social services [18]. Further research into these phenomena as they pertain to CS is particularly salient in the context of growing CS rates.

This study sought to utilize a mixed-methods approach consisting of quantitative data collection from birthing parents of infants who were treated for CS at a large Chicago-area tertiary care center and in-depth qualitative interviews with a subset of these birthing parents and local prenatal providers. In doing so, we aimed to identify causes behind the gaps in the CS prevention cascade and further characterize the factors underlying the rise of CS in Chicago.

## METHODS

### Study Design

#### Eligibility Criteria

Semistructured interviews were conducted with 2 cohorts: (1) birthing parents who delivered infants with CS at a major academic medical institution (AMI) and (2) prenatal providers who either worked at the AMI or within a prenatal clinic that the birthing parents received care from. The AMI is a private academic hospital with 811 beds on the south side of Chicago, Illinois, with 2716 deliveries reported in 2023. The catchment area includes most of the Chicagoland area and Northwest Indiana, and both public insurance and private insurance are accepted, along with participation in various government programs to provide care for low-income populations. While all the women eligible for recruitment delivered at the AMI, they received care from 30 different private and public prenatal clinics. For the first cohort, a purposive sample of birthing parents who delivered between January 1, 2021, and December 31, 2023, was selected using retrospective data abstraction from EHR data. For the second cohort, a purposive sample was utilized through EHR selection, including obstetric/gynecology attending physicians, obstetric/gynecology fellow and resident doctors, nurse midwives, other prenatal advanced practitioners, family medicine providers, and prenatal adolescent medicine providers.

#### Data Collection

We conducted 30–45-minute, semistructured interviews, all of which were performed virtually over the telephone or via teleconferencing technology. Informed consent was obtained from all participants with the study explained in detail, and understanding was confirmed with verbal reciprocation of the details of the study from the participants. The basis for the interview questions was elaborated upon based on both components of the CS prevention cascade, specifically related to entry to care, retention to care, and linkage to care [12], and approaches suggested by CDC clinical care guidelines (Supplementary Table A) [3]. For the first cohort, interview questions addressed the birthing parent's experiences within the prenatal clinic setting and the inpatient peripartum delivery setting, both related to and not related to their syphilis diagnoses, their social network support systems, and their experiences with disclosing their diagnosis to said support systems. Similar questions were presented for the second cohort, in addition to their opinions on potential causes of the rise of CS, barriers to care within and outside of their personal clinical settings, and potential solutions to help reduce the incidence of CS. Stigma was assessed through the validated definitions described by The Health Stigma and Discrimination Network [19]. The interview protocol was created and pilot-tested with clinicians not associated with the project to ensure the relevance and clarity of questions, the usefulness of probes, optimal sequencing of domains, and to enhance reliability among 4 trained and experienced interviewers. For the qualitative analysis, raw data consisted of transcripts of the recorded interviews, utilizing a professional HIPPA-compliant transcription company [20]. These data were subsequently reviewed by the interviewer for completeness and accuracy.

Quantitative data were collected utilizing retrospective data collection through EHR abstraction of birthing parents who delivered infants with CS between 2012 and 2023. Demographics captured included age, race, ethnicity, HIV status, income, medical insurance at the time of the visit, type of CS diagnosis per CDC Case Definition (less likely, possible, symptomatic/highly probable) [1], and gestational age of infant at time of delivery. Prenatal care was categorized as within the academic medical institution (AMI), outside of the institution, or no prenatal care documented. All women had positive treponemal antibody results with reflex to nontreponemal results (RPRs). Additionally, RPRs were collected through EHRs from both the birthing parent and the infant. Among the infants with CS, treatment was defined as no treatment (no penicillin received during pregnancy), insufficient treatment (the birthing parent had either late latent syphilis or unknown acquisition and either received 1 or 2 doses of IM benzathine penicillin when 3 were required or their doses were spread too far apart), or incomplete treatment/treatment failure (women who received their treatment before the pregnancy or during the pregnancy but had RPR levels that did not show adequate reduction in titer levels per CDC guidelines, or the patient was suspected to be reinfected).

#### Statistical Analysis

The study reports descriptive statistics among our sample. Syphilis treatment outcomes were compared using the *t* test and chi-square tests for continuous and categorical predictors, respectively. From these univariate analyses, ordered regression models were performed to indicate the magnitude of the association of the significant predictors with syphilis treatment. Analyses were adjusted for maternal age, medical insurance, and infant treatment. *P* values of ≤.05 were considered significant. All quantitative analyses were performed in STATA, version 17 [21].

For qualitative analyses, text coding was guided by an initial codebook that was further developed and amended during data review. There were 3 trained reviewers who independently reviewed the transcripts and coded them using qualitative analytic software with Dedoose [22]. To ensure internal consistency among reviewers, coding for the first 10% of the transcripts was performed by all 3 coders until an optimal inter-rater reliability kappa score was achieved (0.872; with a standard error of 0.032; 95% CI, 0.80928–0.93472) [23]. Coders met weekly to discuss questions and uncertain code assignments and proposed codebook additions during this coding phase. A trained qualitative analyst then reviewed all coded transcripts for salient themes across interviews. Themes were discussed with the coders, aiding the determination of thematic saturation [24]. This study was approved by the Institutional Review Board.

## RESULTS

Table 1 displays the demographics and syphilis treatment outcomes of the retrospective EHR review. There was a total of 179 women who met eligibility criteria. The mean age (SD) was 26.3 (5.5) years; participants were predominately Black (87.7%), had Medicaid insurance (81.6%), and received care outside of the AMI (60.3%). Of note, 11.2% of patients did not have any documented prenatal care noted in the EHR. From this sample (n = 179), 51.3% completed full treatment, 23.4% partial treatment, and 25.1% received no treatment.

**Table 1.** Demographics and Descriptive Statistics of Birthing Parents who Delivered Infants With Congenital Syphilis From 2012 to 2023, University of Chicago Medical Center, Chicago, IL (n = 179)

	No Treatment (n = 43)		Partial Treatment (n = 42)^a^		Full Treatment (n = 92)		Total (n = 179)		*P* Value
	No.	%	No.	%	No.	%	No.	%	
Age, mean (SD), y	26.2 (5.8)		26.7 (5.7)		26.2 (5.3)		26.3 (5.5)		.88
Age (categorical)									
≤24 y	19	44.2	15	39.5	38	43.2	72	42.6	.96
25–29 y	14	32.6	12	31.6	25	28.4	51	30.2	
≥30 y	10	23.3	11	30.0	25	28.4	46	27.2	
Race^b^									.73
Black	42	93.3	38	90.5	77	83.7	157	87.7	
White	1	2.2	2	4.8	4	4.4	7	3.9	
Other	0	0.0	1	2.4	3	3.3	4	2.2	
Ethnicity^c^									.74
Hispanic	2	4.7	1	2.4	6	6.7	9	5.2	
Non-Hispanic	41	95.4	41	97.6	83	93.3	165	94.8	
Payor									.11
Medicaid	31	68.9	37	88.1	78	84.8	146	81.6	
Private	3	6.7	0	0.0	5	5.4	8	4.5	
Other	1	2.2	0	0.0	1	1.1	2	1.1	
None	10	22.2	5	11.9	8	8.7	23	12.9	
HIV positive	0	46.7	1	2.4	1	1.1	2	1.1	.48
Prenatal care^d^									
Within institution	7	15.5	12	28.6	32	34.8	51	28.5	<.01*
Outside of institution	26	57.8	24	57.1	58	63.0	108	60.3	
None	12	26.7	6	14.3	2	2.2	20	11.2	
Type of CS									<.01*
Less likely	4	8.9	1	2.4	42	45.7	47	26.3	
Possible	39	86.7	39	92.9	44	47.8	122	68.2	
Symptomatic/proven	2	4.4	2	4.8	6	6.5	10	5.6	
Maternal RPR titers									
1st trimester RPR, mean (SD)	0.5 (1.0)		14.1 (16.4)		34.8 (82.3)		29.0 (72.4)		.45
Has a 1st trimester RPR	4	8.9	15	35.7	58	63.0	77	43.0	<.01*
3rd trimester RPR, mean (SD)	73.8 (216.2)		27.4 (79.7)		17.7 (37.4)		28.8 (98.5)		.04*
Has a 3rd trimester RPR	21	46.7	0	0.0	0	0.0	21	11.7	−
Infant RPR titers									
Infant RPR, mean (SD)	5.9 (14.3)		5.6 (10.5)		6.2 (14.8)		6.0 (13.7)		.97
No infant RPR	2	4.4	0	0.0	1	1.1	3	1.7	.29
Infant treatment									
1 d	14	31.1	4	9.5	53	57.6	71	39.0	<.01*
>1 d	31	68.9	38	90.5	39	42.4	108	60.3	

Abbreviations: CDC, Centers for Disease Control and Prevention; CS, congenital syphilis; RPR, reflex to nontreponemal results.

*P values of analyses with a value ≤.05 are displayed in bold.

^a^Partial treatment is defined as a pregnant person who was diagnosed with late latent or unknown timing of syphilis acquisition and only completed 1 or 2 doses of the CDC-recommended 3-dose penicillin regimen.

^b^Race (n = 11).

^c^Ethnicity (n = 5).

^d^Site (n = 3).

Prenatal care was significantly different among treatment groups, with a low proportion of no treatment among those within the institution and the highest proportion of full treatment for patients who received care outside of the institution. There was a significant relationship between type of CS and treatment, with lower odds of those who had a symptomatic/proven type of CS receiving full treatment compared with partial or no treatment than patients who had a less common type of CS (Table 2).

**Table 2.** Regression Models to Indicate the Magnitude of the Association of Significant Predictors With Syphilis Treatment

	No Treatment (n = 45)	Partial Treatment (n = 42)	Full Treatment (n = 92)	Proportional Odds Ratio^a^ (95% CI)	*P* Value
	No. (%)	No. (%)	No. (%)		
Prenatal care					
Within institution	7 (13.7)	12 (23.5)	32 (62.8)	1.26 (0.62–2.56)	.524
Outside of institution	26 (24.1)	24 (22.2)	58 (53.7)	Reference	
Type of CS					
Symptomatic/proven	42 (45.7)	44 (47.8)	6 (6.5)	0.08 (0.02–0.28)	**<.01**
Possible	1 (2.4)	39 (92.9)	2 (4.8)	0.21 (0.94–1.05)	.09
Less likely	4 (8.9)	39 (86.7)	2 (4.4)	Reference	
Maternal RPR levels					
Has a 1st trimester RPR	4 (5.2)	15 (19.5)	58 (75.3)	5.49 (2.73–11.1)	**<.01**
3rd trimester RPR, mean (SD)	73.8 (216.2)	27.4 (79.7)	17.7 (37.4)	1.00 (0.99–1.00)	.07
Infant treatment					
1 d	14 (19.7)	4 (5.6)	53 (74.7)	3.59 (1.83–7.05)	**<.01**
>1 d	31 (28.7)	38 (35.2)	39 (36.1)	Reference	

Abbreviations: CS, congenital syphilis; RPR, reflex to nontreponemal results.

Statistical significance set a priori for *P* values ≤ .05 in the logistic regression. Statistically significant outcomes are noted in bold.

^a^Adjusted models controlled for maternal age, insurance, and infant treatment.

There were 5.49 (95% CI, 2.73–11.06) greater odds of receiving full treatment if maternal RPR was collected in the first trimester compared with those who did not have RPR collected (Table 2). Third-trimester maternal RPR levels were different across groups, with a mean (SD) of 73.8 (216.2) among those with no treatment vs 27.4 (79.7) in those with partial treatment vs 17.7 (37.4) in those with no treatment (*P* = .04), but this finding was not evident in the adjusted model after controlling for age, insurance, and infant treatment. Infants who received only 1 day of treatment had 3.59 (95% CI, 1.83–7.05) greater the odds of the birthing parent receiving full treatment than partial or none compared with infants who received treatment for >1 day.

For the qualitative aim of this research, we enrolled 10 providers and 7 birthing parents who participated in in-depth interviews. A sample of quotes with associated themes is shown in Table 3.

**Table 3.** Qualitative Themes and Additional Corresponding Quotations From Participants

Themes	Quotes
Internal & external barriers to care	Provider 3: “But I know that the…population faces significant barriers to care, and transportation to the healthcare system, language barriers, all the regular things that would affect any part of their care.“
	Provider 4: “Transportation, childcare because they often have other kids, being able to take off work, all those kind of factors. They all feel like social stuff to me…. Yeah. I just think there's a lot of logistic—between where the clinic is located and the logistical things that the patient has to do to—this isn't syphilis, but to get [inaudible], they have to go across the street and come back. And there's not convenient hours.”
	Provider 5: “Because they’re homeless and they don't have an address, or they do not have a phone, or they’re coming to Illinois and they’re leaving.”
	Birthing Parent 6: “I was able to [go to clinic], but I just chose not to due to being homeless and being afraid that instead of getting the right care or the help that I needed, it was just going to go bad.”
Stigma	Provider 8: “I think that sometimes there's a stigma that can happen with any sexually transmitted disease, so patients maybe not wanting to go to some of those locations or wanting to express those things to their partner or otherwise.”
	Birthing Parent 2: “I was honestly so upset and terrified because I was pregnant. So I was so terrified. I was scared that it's going to pass my baby. She's going to have it that she's going to have to live with it. And now we’ll have to explain to her about it and stuff like that.”
	Birthing Parent 4: “My partner freaked out. And I assumed it was my responsibility, you could say, because since we’re not in a committed relationship, I just kind of told him, ‘Look, if it was me that gave it to you, I am so sorry, but you need to get checked…. [I didn't tell family] because of the shame that goes behind it. And I wouldn't want—during that time, I already felt guilty, you could say, that perhaps I caused this to my baby, in a sense.”
	Birthing Parent 6: “They didn't look at me as someone trying to get help. They looked at me as just another person in the street on drugs. So that was one of my fears that they would have taken my baby. So I just chose not—I did my care myself.”
Health literacy & education	Provider 8: “So I think some of that is medical health literacy, having an understanding of that barrier of communication sometimes between systems as patients show up to multiple centers or areas that don't communicate or, for example, if they’re truly immigrant to the US, may have received it in a different country or elsewhere, may or may not have records of it, or that kind of insider understanding of like, ‘Oh, I just received an antibiotic, but I don't remember how much or how many.’”
	Provider 10: “And then the other thing is I think they may not have received a lot of counseling about congenital syphilis, in general, to really understand why we’re so harping on getting treatment.”
	Birthing Parent 2: “They basically were saying I may have—one thing they were telling me—I’m trying to remember. It was so long. What they were saying—dang, I forgot what they were saying.”
	Birthing Parent 5: “Very brief. Like I said, I didn't take it so serious because I was told we’re okay…. Do I need to get more education on it? Yes, absolutely.”
Communication	Provider 10: “I think they may not have received a lot of counseling about congenital syphilis, in general, to really understand why we’re so harping on getting treatment.”
	Birthing Parent 1: “I just felt like they didn't care. That's just one concern I have to say about [clinic]. I just feel like they really didn't care. So that's just my opinion for them.”
	Birthing Parent 3: “It's terrible, and I let them know. The communication was completely terrible. Even when it came to when I was admitted in there.”
Appointment burden	Provider 4: “I think there are a lot of social factors that can prohibit you from getting to appointments depending on where you have to go. Three appointments in three weeks is a lot.”
	Provider 7: “But in our practice, so a lot of times in our OB triage area, people will get a syphilis test, and then we can't find them to get any penicillin. Those are patients that aren't in care in our clinic. And then patients in care in our clinic, we usually can get them back for one shot, but it can be difficult getting them back.”
	Birthing Parent 1: “Having to be there at a certain time and then end up not being able to make it on time and knowing that I had to catch a bus from a different distance, which made it so difficult, especially if I wasn't in a Lyft or Uber or whatever.”

Abbreviation: OB, obstetrics.

### Providers

As far as treatment for CS, the majority of providers mentioned instances where their patients only received a partial course of penicillin treatment. Some providers described how some of their patients receive full treatment courses for syphilis, but some may not due to various barriers to care, and other providers spoke to having to re-treat birthing parents who were only able to receive partial courses of treatment. The most common barrier to care and successful treatment of CS that was cited by every provider in this sample was transportation. Specifically, barriers related to transportation included low car ownership, prolonged commutes either on public or private transportation, and financial restrictions related to public transporation costs or gasoline costs. Other barriers included challenges with being unable to communicate with patients beyond an established appointment, gaps in patient understanding or knowledge around CS contraction and treatment, appointment burden for patients, and stigma related to the CS diagnosis.

The majority of providers said that they have noticed an increase in syphilis among their birthing patient panels, attributing this increase to a multitude of additional factors such as a longstanding emphasis on HIV for perinatal transmission, decreases in sexually transmitted infection (STI) testing and treatment during the coronavirus disease 2019 (COVID-19) pandemic, less funding for STI clinics, changes in screening guidelines over the years, and increasing migrant and refugee populations who are seen in clinics.

### Birthing Parents

Almost all birthing parents (86%, n = 6) in our sample reported positive experiences receiving care at the AMI. Common factors that contributed to their positive experiences included helpful doctors and staff and frequent check-ins with the birthing parents and making sure their needs were met during their visit. Birthing parents also discussed feeling welcomed and safe while receiving care at the AMI and appreciated being treated with respect and without judgment by providers and staff. One of the birthing parents had a poor experience postdelivery due to poor interdepartmental communication between the birthing parent's care team and her baby's neonatal intensive care unit care team. While she described the communication with her infant's care team as “completely terrible,” she would still consider receiving care through the AMI, particularly because her insurance would be accepted at this facility.

Some birthing parents found out about their CS infection early in pregnancy, while others did not learn about the infection until late in pregnancy or at delivery. There was a myriad of concerns that emerged regarding the education that these birthing parents received around CS in all clinical settings. One birthing parent felt that the staff at her prenatal clinic “just didn't care,” and another reported not receiving any education from her prenatal provider when she was diagnosed, prompting independent research on her part. One birthing parent wished that the doctor had elaborated more on the disease when she was diagnosed. Another received insufficient education around CS before moving to Chicago, and she was unable to keep up with her penicillin treatment until she established care at the AMI due to being unstably housed in California, as well as experiences with stigma and mistrust of the medical system.

Birthing parents also reflected on how they felt when they found out about their diagnosis, and most expressed feelings of shame, guilt, embarrassment, and fear related to having CS, requiring penicillin treatment for both birthing parents and their babies, and about the potential for infection in their babies postdelivery. However, they were willing to go through penicillin treatment for themselves and their infants in the end because they wanted what was best for them.

Shame was also closely related to birthing parents’ willingness to disclose their diagnosis to others, as many reported only telling 1–2 people in their networks, often sexual partners or close family members and friends, due to feeling embarrassed by the diagnosis. One birthing parent did not disclose her diagnosis to anyone because she felt that it was a personal problem and “would have been a big issue” if her friends or family found out about her diagnosis. Two birthing parents only disclosed the diagnosis to their sexual partners because it was “not anyone else's business.” Some birthing parents had supportive family members or friends who offered help with attending appointments or emotional support after disclosure, while others experienced a negative reaction, some from sexual partners who blamed the birthing parent for contracting CS.

Multiple birthing parents reported various barriers to obtaining testing and treatment for CS, including limited transportation to prenatal appointments, appointment burden and lack of timely appointment availability, and embarrassment or stigma around the diagnosis of CS. Multiple mothers reported restricted access to cars, often shared with other friends and family members, prolonged commutes when taking public transportation, and the financial strain of high gasoline costs and public transportation fees. Less common but equally crucial barriers that came up were unstable housing, financial burden, and poor communication between birthing parents and baby care teams postdelivery.

## DISCUSSION

**Figure 1.**
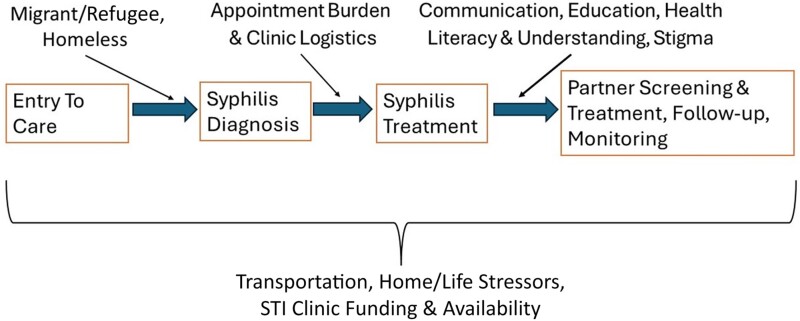
Qualitative themes and their impact on the maternal syphilis care continuum and congenital syphilis prevention cascade. Abbreviation: STI, sexually transmitted infection.

This mixed-methods study utilizing retrospective quantitative analysis of birthing parents who birthed infants with CS and qualitative methods through a semistructured interview process of prenatal providers and birthing parents who birth infants with CS found numerous perspectives and factors that may explain the rise of CS along various stages of the syphilis care continuum and CS prevention cascade (Figure 1, Table 3) [12]. Notably, barriers elicited during the qualitative interviews included insufficient penicillin uptake, limited transportation, poor communication between providers and patients, gaps in patient understanding or knowledge around CS contraction and treatment, missed appointments, appointment burden for patients, life stressors of patients, housing instability, childcare difficulties, and stigma related to the CS diagnosis. Additional factors related to this included a longstanding emphasis on HIV for perinatal transmission detracting focus from other STIs, decreases in STI testing and treatment during the COVID-19 pandemic, less funding for STI clinics, changes in screening guidelines over the years, and increasing migrant and refugee populations who are seen in clinics.

The quantitative data revealed that among patients who received prenatal care outside the institution there was a higher proportion of those receiving full treatment vs the combined partial and no treatment than among patients who had no prenatal care, with similar findings for patients who received care within the AMI. With regards to maternal RPR levels, the study found greater odds of birthing parents who received first trimester RPRs receiving full treatment compared with partial or no treatment than those who had no maternal RPR levels during the first trimester, but not in the third trimester.

The findings further support previous work revealing the impact of financial limitations and issues related to insurance as factors that may impact the rise of CS [12, 15–17]. Additionally, it is noted in the retrospective cohort analysis that there was insufficient prenatal syphilis testing and insufficient treatment during pregnancy, which was described previously [3, 17]. Another study was able to identify more occult findings through the qualitative methods, including the impact of stigma related to the diagnosis and management of both maternal and CS, and expand on issues related to challenges in testing and treatment including discretionary testing procedures, delays in screening results, and treatment referral issues [17]. Specific forms of stigma identified included internalized (self) stigma among the birthing parents, external stigma felt from partners, family, and health care employees (structural), and public stigma [25, 26]. The study was able to glean additional insights including a significant emphasis on transportation barriers and home life stressors of the birthing parents. A focus on minoritized and under-resourced community members, including American Indian/American Natives and Black Americans, in whom we are seeing the largest epidemic [1], is critically important in order for health equity improvements to be obtained, particularly in the context of CS prevention and management.

Potential next steps based on the results of the study could include boosted prenatal clinic outreach interventions, alleviations in appointment burden for patients, and improved communication between clinics and patients. Given that transportation restrictions and limitations were one of the greatest themes noted through all the interviews, additional interventions related to travel assistance programs or mobile penicillin delivery services could also be considered and have shown promising results for other chronic medical conditions and to reach marginalized and disenfranchised populations [27, 28]. Given the reported disclosure stigma among birthing parents, there could also be clinical and public health outreach interventions to seek to improve partner notification, screening, and treatment to assist in reducing onward syphilis transmission [29]. Peer education and sensitization training have also been shown to reduce systemic sexual stigma toward men who have sex with men with HIV [30], and these strategies could be translated to other sexually transmitted infections including syphilis [31]. Additionally, structural interventions focused at reducing internalized stigma among women with HIV have been successfully implemented and could be replicated to other STIs, such as syphilis [32]. The increased implementation of doxycycline postexposure prophylaxis (DoxyPEP) among high-risk individuals may also contribute to reductions of syphilis in the communities and alleviate the burden of the disease [33].

The study had multiple strengths that enhanced the impact of the study. The survey team engaged a group of prenatal providers and birthing parents in the Midwestern United States that had not been previously described. The data help support other regional and nationwide studies that demonstrate the primary gaps in the syphilis care continuum and CS prevention cascade that are focused on entry into care, retention in care, and assessing linkage gaps in care [12]. However, we were able to elicit unobserved factors such as issues with transportation, internalized and systemic stigma, appointment burden, and home stressors that are not easily obtained from the medical record. These findings allow for the ability to directly translate into clinical and public health interventions to help improve prevention of CS.

The study has several limitations that may impact the validity of the study. Limitations related to the qualitative component of the study include the subjectivity of the participants and data analyzers, response bias, the potential for overinterpretation, selection bias, social desirability bias, and recall bias. We attempted to overcome these limitations through rigorous auditing of the coding process, having a third-party qualitative analyst validate the coding, and purposefully sampling all potential women who had delivered at the institution regardless of background. Additionally, we limited the qualitative interview time frame to the last 3 years to reduce recall bias potential. The project involved interviewing prenatal providers in Chicago, Illinois, which may not be generalizable to the training and clinical practices of prenatal providers in other parts of the country or the world. Both with the qualitative interviews and the quantitative retrospective component of the study, the majority of the birthing parents were generally homogenous when it came to race, ethnicity, and payor, which may not be generalizable to other populations of differing demographic backgrounds. All testing that was extracted from the electronic health record was done in the prenatal clinic setting. It is uncertain based off the limitations of the extraction if this was done through rapid tests or if the laboratory tests had to be sent to a separate microbiology laboratory. While we were able to reach thematic saturation among the prenatal providers, an interview total of only 7 birthing parents may alter the results and interpretation, lowering the power. Reasons for the limited availability of birthing parents included inaccurate contact information in the EHR, time constraints of the potential participants, and refusal to participate due to uncertainty of the content of the study or reported mistrust in the medical research processes. Additionally, due to substantial barriers to recruitment noted during the process including inaccurate contact information in the EHR, time availability of potential participants, and mistrust in the medical system, the low number of maternal interviewees at 7 could also affect the findings. Regarding the quantitative analysis, additional limitations included limited external generalizability, missing data or misdocumented data from the EHR, and unobserved factors that may be confounding variables in the analysis.

## CONCLUSIONS

This study found numerous perspectives and factors that may explain the rise of CS along various stages of the syphilis care continuum and CS prevention cascade through in-depth interviews of prenatal providers and birthing parents. It is vital that these results be utilized for downstream research studies and public health initiatives to allow for targeted interventions that may efficiently assist in ending this rising epidemic and prevent potentially devastating downstream effects of this preventable disease.

## Supplementary Data

Supplementary materials are available at *Open Forum Infectious Diseases* online. Consisting of data provided by the authors to benefit the reader, the posted materials are not copyedited and are the sole responsibility of the authors, so questions or comments should be addressed to the corresponding author.

## Supplementary Material

ofae595_Supplementary_Data
