# Supplementary material for: Factors Related to the Rise of Congenital Syphilis From the Perspectives of Prenatal Providers and Birthing Parents in Chicago, IL, USA
Source: Open Forum Infect Dis. 2024 Oct 8;11(10):ofae595. doi: 10.1093/ofid/ofae595 (PMC11521334; doi:10.1093/ofid/ofae595)
Supplement: ofae595_Supplementary_Data [file ofae595_supplementary_data.docx]

**Supplemental Document A – Interview Guides for Qualitative Interviews**

**Interview Guide for Cohort #1 (Mothers whose infants were born with CS)**

1. Tell me about your experience delivering your child at your local hospital/medical center.
2. What made you decide to deliver there?

- What things went well during your time at the hospital?
- What things did not go well during your time?
- Is there anything that could have been improved around your delivery experience?

1. Would you be interested in receiving care again at this location for yourself? Would you recommend receiving care at this location to your friends and family who may become pregnant in the future? Why or why not?
2. After your baby was born, do you recall you and your baby receiving a medicine called penicillin to treat an infection called syphilis?
   1. Did you know about having the infection syphilis while you were pregnant?
3. What sort of education and information was offered to you by your health care provider, while you were pregnant, about the infection syphilis?
   1. What do you remember about your healthcare provider teaching you about syphilis at any point?
4. What was your experience like talking with your health care provider about treatments for syphilis, such as an antibiotic like penicillin?
5. How did it make you feel learning about having the infection, syphilis?
6. How did it make you feel learning that your baby needed penicillin for the infection?
7. Did other people around you know that you or your baby were diagnosed with syphilis or required treatment for syphilis? (If ‘yes’, go to 12a. If ‘no’, go to 12b)
   1. YES: What sort of reaction did your friends or family have to this information?
   2. NO: Were there any particular reasons why your friends or family did not know this information?
8. Was your partner aware of your diagnosis? Of the baby’s diagnosis? Did you partner ever get tested? Why or why not?

**Interview Guide for Cohort #2 (Providers**)

1. Tell me about the sociodemographic characteristics of your patient population within your prenatal practice. Do you see some specific patient populations more than others in your practice?
2. Tell me about the day-to-day logistics of patient care in your clinic.
   1. How many patients are coming into your clinic on a typical day? How many patients are you seeing?
   2. How much time do you have to meet with each patient?
3. Have you noticed an increase in acquired syphilis, including positive RPR tests, among your patient population?
   1. If YES: What do you attribute that increase to?
4. When a patient is diagnosed with syphilis in clinic, what is the process for the patient to receive necessary treatment with intramuscular penicillin?
5. Do you feel all patients in general successfully receive full treatment courses for syphilis? (If no, go to question 5a, if yes, go to question 6)
   1. If NO: Why do you feel those patients did not receive full treatment courses?
6. What barriers to care outside of clinic, if any, do you feel may prevent patients from receiving successful courses of syphilis treatment?
   1. Probe if needed: transportation, insurance, patient motivation, medical mistrust, etc.
7. What barriers to care within your clinic, if any, do you feel may prevent patients from receiving successful courses of syphilis treatment?
   1. Probe if needed: rapport with patients, clinic staffing/availability, etc.
8. What is your professional title in patient care? (e.g., Obstetrics/gynecology MD or DO, Family Medicine MD or DO)
9. How long have you been practicing in your current practice? (e.g., 0-2 years, 3-5 years, 6-8 years, 8-10 years, Greater than 10 years)
10. How long have you been practicing since completion of your training? (e.g., 0-5 years, 5-10 years, 10-15 years, 15-20 years, > 20 years)
